# Virilizing adrenal carcinoma in a 3-year-old boy: A rarity

**DOI:** 10.4103/0971-5851.68851

**Published:** 2010

**Authors:** Suresh Kumar, Punit Tiwari, Ranjeet Kr Das, Anup Kr Kundu

**Affiliations:** *Department of Urology, IPGMER, Kolkata, India*

**Keywords:** *Adrenal carcinoma*, *adrenalectomy*, *virilization*

## Abstract

A 3-year-old boy presented with iso-sexual precocious puberty for 18 months. Radiological images revealed left suprarenal mass and hormonal profile showed markedly increased testosterone and dehydroepiandrosterone-sulfate. The child underwent open adrenalectomy and histopathology revealed adrenocortical carcinoma. At 3 months follow-up, the child is doing well.

## INTRODUCTION

Adrenocortical neoplasm has bimodal age distribution, with its peak in the first and fifth decades. Adrenocortical neoplasms in children are uncommon, comprising 6% of all adrenal neoplasms and 0.3% of all pediatric neoplasms[1] Virilizing adrenal carcinoma is a very rare disease, and the estimated incidence is 1 case/1.7 million.[2] It is more likely to be found in girls than in boys. We report a 3-year-old boy who presented with iso-sexual precocious puberty with virilizing adrenal cancer.

## CASE REPORT

A 3-year-old boy presented with enlargement of the penis and pubic hair for 18 months and deepening of voice and axillary hair for 6 months. On examination, the child was otherwise healthy and normotensive Pubic hairs, axillary hairs and hairs in the beard area and upper lip were present. Stretched penile length was 9.8 cm. Bilateral testes were normally descended and normal in size. There were no skin spots and no evidence of features of Cushing syndrome. No lump was palpable on abdominal examination. Spine and digital rectal examinations were unremarkable.

His hemoglobin was 14.0 g% and blood biochemistry reports were normal, except serum alkaline phosphatase which was 939 U/l. Serum electrolytes were Na: 137 meq/l and K: 4.2 meq/l. Chest X-ray was normal. Hormone profile of the patient is summarized in Table 1.

**Table 1:** Pre-operative hormonal profile

Serum hormone	Normal range	Preoperative level
Serum testosterone (ng/ml)	0.12–0.32	9.7
Serum DHEA-S (μg/dl)	0.47–29.4	>1,000
Serum FSH (mlU/ml)	0.2–3.8	0.15
Serum β HCG (mlU/ml)	Up to 0.6	<0.6
Serum Cortisol; 8.0 am (μg/dl)	4.3–22.4	12.68

**Figure 1:**
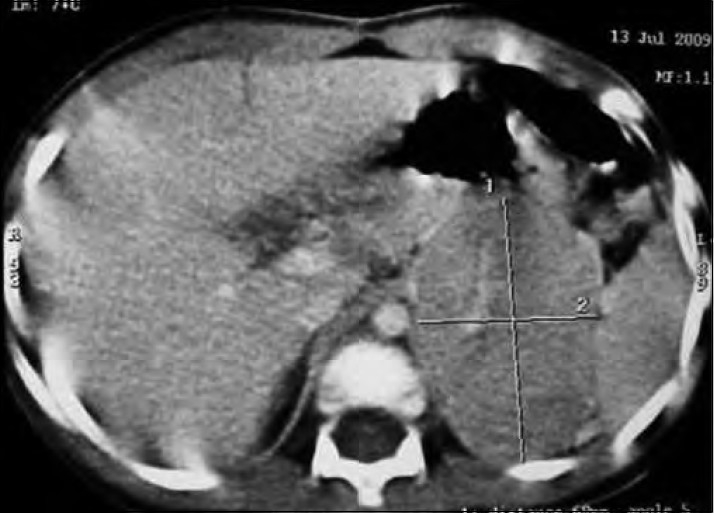
Contrast enhanced computed tomography-left suprarenal mass showing rim-like enhancing capsule

Ultrasonography of the abdomen revealed left adrenal solid mass with few speculated calcifications. Contrast enhanced computed tomography (CT)-whole abdomen [Figure 1] showed a large 68×47 mm soft tissue density mass in the left suprarenal region, showing homogenous contrast enhancement, displacing the left kidney downward with a thin rim-like enhancing capsule around the mass.

The child underwent left open adrenalectomy via subcostal transperitoneal approach. The postoperative period remained uneventful.

**Figure 2:**
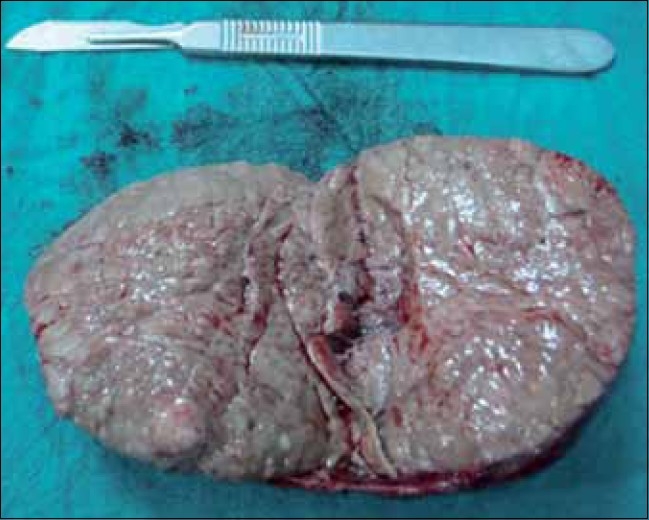
Cut-section: variegated pattern with intratumoral nodules

**Figure 3:**
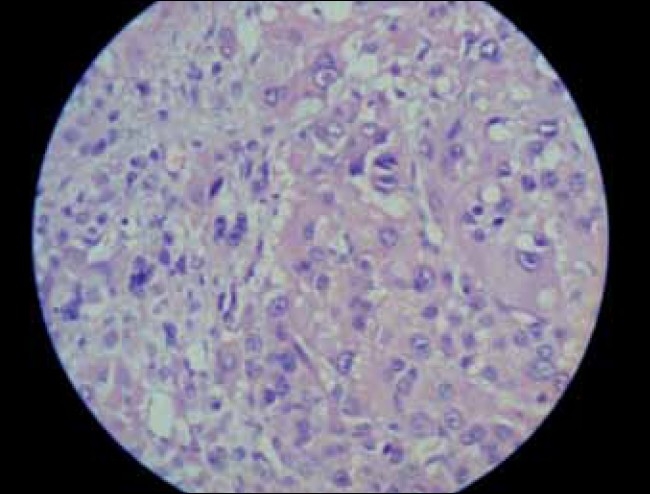
Marked nuclear pleomorphism, tumor giant cells and high mitotic figures (high magnification)

Histopathology revealed a well-circumscribed, encapsulated nodular tumor (6.7 cm) in its greatest axis, weighing 125g. The cut-section showed a variegated pattern, with soft and friable intratumoral nodules with areas of hemorrhage and necrosis [Figure 2]. Microscopically, it consisted of large polygonal cells with vesicular nuclei and prominent nucleoli, marked nuclear pleomorphism, tumor giant cells with bizarre hyperchromatic nuclei and abundant eosinophilic cytoplasm, and mitotic figures were >20/50 hpf [Figure 3]. Two weeks postoperatively, the serum testosterone and DHEA-S returned to normal. At 3 months follow-up, the child is doing well, with normal chest X-ray and ultrasonography of the whole abdomen.

## DISCUSSION

Adrenocortical carcinomas with mixed virilizing and Cushing’s syndrome are typical, but pure virilizing adrenocortical carcinomas are uncommon, comprising 5–10% of the cases in most large series.[3] Virilizing adrenocortical carcinoma causes iso-sexual precocious puberty in boys and virilization in girls. The hormonal profile is assessed by estimating the serum levels of testosterone, DHEA-S, β-HCG, FSH and cortisol and urinary 17-ketosteroid and 17-hydroxycorticosteroid. Most cases with virilization have elevated levels of dehydroepiandrosterone. Elevated testosterone levels are usually attributed to peripheral conversion of adrenal androgens.

Our patient had markedly increased serum testosterone and serum DHEA-S. High concentrations of adrenal androgens, especially DHEA-S, are highly suggestive of a virilizing tumor and contrast-enhanced CT-abdomen localizes the adrenal mass.

Prognosis for benign adrenal tumors is excellent. On the other hand, usual prognosis for adrenocortical carcinoma is generally poor, with an overall 5-year survival of 20–25%. Factors associated with poor prognosis, specifically for virilizing tumors, include incomplete resection, weight >80 g, volume >200 ccm, age >3.5 years at diagnosis, preoperative symptom duration >6 months and marked increase in urinary 17-ketosteroid and 17-hydroxysteroids. Pure virilizing carcinomas in general appear to have a better prognosis than other adrenal carcinomas.[4] In our case, poor prognostic factors were tumor weight 125 g and preoperative symptom duration 18 months.

Bugg *et al*. utilized the modified criteria of Weiss to analyze pediatric adrenocortical tumors.[4] This classification was based on mitotic index, confluent necrosis, atypical mitosis and nuclear grade. According to this classification, our patient had high-grade adrenocortical carcinoma.

Regarding staging, in our case, the tumor was completely resected with a negative margin, weighed 125 g and there was no evidence of metastasis. The abnormal hormone levels returned to normal after surgery and thus our case was labelled as stage 1, high-grade adrenocortical carcinoma.

Hence, it is concluded that a child with virilizing symptoms should be promptly investigated for biochemical profile and radiological imaging. Surgical resection is the treatment of choice. Surgery is the only definitive therapeutic modality in children, which can be curative only if adrenocortical carcinoma is diagnosed early and treated promptly.

Adjuvant chemotherapy is used for metastatic or recurrent disease, but paucity of cases makes its evaluation difficult.[5] The role of radiotherapy is controversial.
